# Single Laboratory Validation of A Ready-to-Use Phosphatase Inhibition Assay for Detection of Okadaic Acid Toxins

**DOI:** 10.3390/toxins4050339

**Published:** 2012-04-30

**Authors:** Henry G. F. Smienk, Dolores Calvo, Pedro Razquin, Elena Domínguez, Luis Mata

**Affiliations:** Zeu-Inmunotec, Polígono PLAZA, C/Bari 25 Dpdo, 50197, Zaragoza, Spain; Email: hsmienk@zeulab.com (H.G.F.S.); dcalvo@zeulab.com (D.C.); prazquin@zeulab.com (P.R.); lmata@zeulab.com (L.M.)

**Keywords:** protein phosphatase inhibition assay (PPIA), protein phosphatase 2A (PP2A), validation, okadaic acid (OA), diarrheic shellfish poisoning (DSP)

## Abstract

A phosphatase inhibition assay for detection of okadaic acid (OA) toxins in shellfish, OkaTest, was single laboratory validated according to international recognized guidelines (AOAC, EURACHEM). Special emphasis was placed on the ruggedness of the method and stability of the components. All reagents were stable for more than 6 months and the method was highly robust under normal laboratory conditions. The limit of detection and quantification were 44 and 56 µg/kg, respectively; both below the European legal limit of 160 µg/kg. The repeatability was evaluated with 2 naturally contaminated samples. The relative standard deviation (RSD) calculated was 1.4% at a level of 276 µg/kg and 3.9% at 124 µg/kg. Intermediate precision was estimated by testing 10 different samples (mussel and scallop) on three different days and ranged between 2.4 and 9.5%. The IC_50_ values of the phosphatase used in this assay were determined for OA (1.2 nM), DTX-1 (1.6 nM) and DTX-2 (1.2 nM). The accuracy of the method was estimated by recovery testing for OA (mussel, 78–101%; king scallop, 98–114%), DTX-1 (king scallop, 79–102%) and DTX-2 (king scallop, 93%). Finally, the method was qualitatively compared to the mouse bioassay and LC-MS/MS.

## 1. Introduction

Diarrheic shellfish poisoning (DSP) is a consequence of the ingestion of a series of lipophilic toxins produced by dinoflagellates that can be present in shellfish for human consumption. These lipophilic toxins can be subdivided into four groups: the okadaic acid group (OA-toxins) including the dinophysistoxins (DTX), the pectenotoxin group (PTX), the yessotoxin group (YTX) and finally the azaspiracids (AZA). Only the OA-toxins and AZA are known to cause gastrointestinal problems [1,2]. For many years the mouse bioassay (MBA) has been the official method of detection for lipophilic toxins in the European Union [3], but with the publication of Commission Regulation (EU) No. 15/2011 [4], LC-MS/MS has become the reference method for their determination. This regulation also states that alternative or complementary methods can be used as long as an equivalent level of public health protection is provided, and the method performance criteria stipulated by the European Union Reference Laboratory on Marine Biotoxins (EU-RLMB) are fulfilled. Such methods should be intra-laboratory validated and successfully tested under a recognized proficiency test scheme. 

Protein phosphatase inhibition assays (PPIA) have been identified for a long time as an alternative for the detection of OA-toxins, as ser/thr phosphatases are known to be their natural target [5,6]. As such, a validated phosphatase inhibition assay can be very useful in lipophilic toxin detection, complementary to the more complex, expensive and time consuming LC-MS/MS; or as an alternative when only OA-toxins are present in the samples. Different laboratories have developed in-house PPIA with good qualifications, using colorimetric or fluorimetric substrates to monitor enzyme inhibition. [7,8,9,10,11,12]. A collaborative study was also performed with a fluorimetric assay [13]. However, specific equipment, not often available in routine testing laboratories, makes difficult the use of fluorimetric assays for monitoring purposes. Besides, fluorimetric substrates are less stable than colorimetric ones and therefore less appropriate for ready-to-use kits. A standardized commercial test based on PPIA has not been available until recently. In this paper, we present a single laboratory validation of a commercial colorimetric PP2A assay (OkaTest) for the determination of OA-toxins in bivalve mollusks.

## 2. Materials and Methods

### 2.1. Reagents and Equipment

OkaTest kit (formerly Toxiline-DSP): The kit includes a 96-well microtiter plate, four vials of lyophilized protein phosphatase 2A (PP2A), purified from human red blood cells, five OA standards (0.5, 0.8, 1.2, 1.8 and 2.8 nM) prepared from the OA Certified Reference Material (NRC CRM-OA-c, NRC-CNRC, Institute for Marine Biosciences), a liquid chromogenic substrate (p-Nitrophenyl phosphate), phosphatase dilution buffer and buffer solution. 

Other reagents not included in the OkaTest kit: Methanol (Reagent grade, Carlo Erba), HCl (Reagent grade, 37% v/v, Carlo Erba), NaOH (Reagent grade, Scharlau), de-ionized water (type II, ISO 3696), certified Reference Materials (NRC CRM-DSP-MUS-b, NRC CRM-OA-c, NRC-CNRC, Institute for Marine Biosciences), DTX-1 (042-28661, Wako) and DTX2 (00-DTX2, Cifga).

Equipment: Ultra homogenizer (IKA werken), a water bath at 76 ± 2 °C (Raypa), a FX-incubator at 30 °C ± 2 °C (ZEU-INMUNOTEC), a microplate absorbance reader (405 nm ± 10 nm wavelength filter, Multiskan RC, Thermo-Labsystems), roller mixer, centrifuge, micropipettes, graduated 50 mL centrifuge tubes and laboratory glassware.

### 2.2. Sample Preparation

Market samples were thoroughly washed, the whole mollusk tissue recovered from the shell, and then blended. Portions of 5 ± 0.1 g were prepared and used for fresh testing, or stored frozen (below −15 °C) for future analysis. The portions were extracted by adding 25 mL of methanol (100% v/v) and mixing with a vortex for 2 min. The methanolic extract was separated by centrifugation for 10 min. at 2000 × g. To perform the hydrolysis, 640 µL of the methanolic extract and 100 µL of 3 N NaOH were mixed and incubated for 40 ± 1 min. at 76 ± 1 °C. To stop the reaction, 80 µL of HCl were added and sample preparation buffer used to make up a final volume of 20 mL. For non-hydrolyzed samples, 640 µL of methanolic extract were diluted up to 20 mL with sample preparation buffer. Hydrolysis was carried out in most samples unless otherwise specified.

### 2.3. Assay Procedure

The phosphatase solution was prepared by adding 2 mL of dilution buffer to each vial of lyophilized PP2A. To assure full hydration of the lyophilized enzyme, it was mixed gently for 1 h ± 5 min. at room temperature (22 °C ± 2 °C) on a roller mixer. Then, 50 µL of samples or ready-to-use OA standards (0.5, 0.8, 1.2, 1.8 and 2.8 nM), and 70 µL of the prepared phosphatase solution were added in duplicate to a microwell plate. This mixture was equilibrated in an incubator for 20 ± 2 min. at 30 °C. Finally, 90 µL of the chromogenic substrate were added to each well and incubated for 30 ± 2 min. at 30 °C. The absorbance was read at 405 nm.

### 2.4. Calculations

The results were calculated from a standard curve by plotting the absorbance values in a linear *y axis* and the concentration of OA in a logarithmic *x axis*, and using a logarithmic fitting. As an acceptability criterion for the assay, the Pearson correlation coefficient *r*^2^ had to be greater than or equal to 0.96. The OA concentration contained in the sample was then calculated using the following equation: 

            *x* = EXP (*y* – b)/a

where *x* is the OA concentration in the sample (*C*_s_) and *y* the absorbance of the sample. 

The OA-toxin concentration in shellfish tissue was calculated as follows: 

           *C*_t_ (µg/kg) = (*C*_s_ (nM) × FD × *MW* (g/mol) × *V*_e_ (L))/*M*_t_ (g)

where *C*_t_ is the toxin concentration in tissue, expressed as equivalents of OA, *FD* is the methanolic extract dilution factor (31.25), *MW* is the OA molecular weight = 805, *V*_e_ is the methanolic extract volume (0.025 L), *M*_t_ is the tissue weight (5 g). 

Samples with an OA concentration falling outside the working range (<0.5 nM or >2.8 nM) will be reported as <63 µg/kg (or <0.5 nM) or >352 µg/kg (or >2.8 nM), respectively.

### 2.5. Ruggedness Testing

The ruggedness testing was performed by introducing changes in the procedure and determining the effects on the sample quantification [14]. The variations used were chosen according to the values expected under normal laboratory conditions.

### 2.6. Spiking Procedure

Samples were spiked with OA Certified Reference Calibration Solution (NRC CRM-OA-c). The reference solution was prediluted to 2 µM in sample buffer and added accordingly. No Certified Reference Materials were available for DTX-1 and DTX-2 at the time of the performance testing. These toxins were first dissolved in methanol and diluted to 2 µM in sample buffer before adding to the samples.

A Certified Reference Material (NRC CRM-DSP-MUS-b) was also tested. However, the certified concentration of this material is far above the working range of the assay and the sample had to be diluted with blank mussel or king scallop. To do this, an amount of reference material was added as precisely as possible to 50 mL tubes, and weighed. The blank material was added on top and the mixture weighed again. Then, the amount of the mussel reference material per sample was calculated. This value was used as the theoretical spiked amount. The samples were analyzed with and without hydrolysis, as the reference material was only certified for OA and DTX-1, but ester derivates of the OA-toxins could also be present as indicated in the CRM certificate. The total recovery was calculated according to the AOAC Official methods of analysis [15].

### 2.7. Method Comparison

A method comparison was also carried out with OkaTest, the mouse bioassay (MBA) and LC-MS/MS, using EU harmonized protocols for the last two methods [16,17].

Shellfish samples were previously tested by a third party laboratory using mouse bioassay (MBA) and LC-MS/MS, and kindly donated to do the method comparison.

As MBA is a qualitative method, results obtained by OkaTest and LC-MS/MS were interpreted qualitatively for comparison purposes. Therefore, samples with a concentration ≥160 µg/kg were regarded as positive, while samples with a concentration <160 µg/kg were reported negative.

## 3. Results and Discussion

### 3.1. Calibration of the Assay

**Figure 1:**
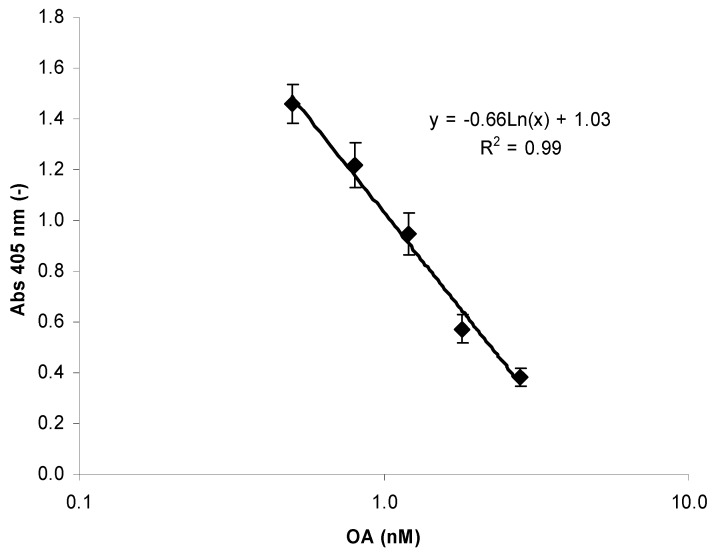
Typical calibration curve of OkaTest produced as the mean of 5 phosphatase batches. The Pearson correlation coefficient (*r*^2^) of the logarithmic fit was >0.96 for each batch. The figure shows the equation and *r*^2^ of the mean. The error bars were calculated as ±1 SD.

The assay is calibrated by five OA standards prepared by dilution from the NRC CRM-OA-c with a concentration between 0.5 and 2.8 nM OA. Following the kits sample preparation (see material and methods), this will result in a working range between 63 and 352 µg/kg. Figure 1 shows a typical calibration curve from 5 different assays using different phosphatase batches. All calibration curves were evaluated according to the Pearson correlation coefficient obtained after a logarithmic fitting procedure (*r*^2^ > 0.96).

The bias introduced by the logarithmic fitting procedure on the calibration curve of the kit was estimated by recalculating the concentration of the OA dilutions using its own standard curve. The relative absolute difference was then calculated as the absolute difference between the theoretical and calculated OA concentration divided by the theoretical OA concentration and multiplied by 100 (Table 1). The best accuracy was found at levels around the regulatory limit (0.8% at 1.2 nM OA standards equals 151 µg OA equivalents/kg mollusk), while below that level (0.5 nM of OA), a 9.0% overestimation was calculated. Only minor deviations were calculated over the legal limit.

**Table 1:** Bias introduced due to the fitting procedure. Relative absolute difference was calculated from mean of 5 standard curves by relating the absolute difference to the theoretical OA concentration.

OA theoretical (nM)	OA calculated (nM)	Relative Absolute Difference
0.50	0.55	9.0%
0.80	0.83	3.8%
1.20	1.21	0.8%
1.80	1.78	1.1%
2.80	2.73	2.5%

### 3.2. Stability and Homogeneity of the Components

**Figure 2:**
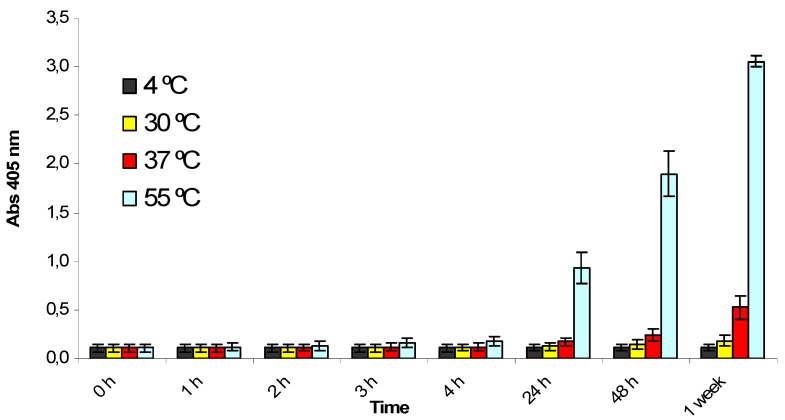
Study of the temperature stability for the ready-to-use chromogenic substrate (p-Nitrophenyl phosphate). Absorbance at 405 nm was measured at different times and temperatures. Assays were performed in triplicate. The error bars were calculated as ±1 SD.

The stability and homogeneity of the critical components of the kit were studied by combining a real time and accelerated study design. Water soluble buffers such as the phosphatase dilution solution and the sample buffer were considered less critical, as sufficient internal know-how was available for these components and no stability problems were expected. Other components, such as the ready-to-use chromogenic substrate, the PP2A or the OA standards, were specially developed for the phosphatase inhibition assay and were more extensively tested. Reagents were normally analyzed within the assay system or by performing specific tests depending on their particular characteristics. The ready-to-use substrate performed correctly in the OkaTest assay when stored for a year at temperatures between 2 and 15 °C (results not shown), as the background absorbance remained acceptable (below 0.3 absorbance units). However, accelerated studies showed that the substrate is sensitive to higher temperatures (Figure 2). After 24 h at 55 °C, the substrate was strongly hydrolyzed and after 1 week at 37 °C the absorbance of the substrate was above 0.6. Nevertheless, these results indicate that although the hydrolysis rate increases with temperature, it is very stable at temperatures below 15 °C and no problems should be expected under normal conditions of usage and storage.

The OA standards and the PP2A were estimated the most critical components, as their quantity and quality establish the working range and, to a great extent, the ruggedness of the assay. The enzyme quantity determines the amount of analyte that is needed for inhibition, while the enzyme quality assures the amount of product formed per time unit [18]. Likewise, the lack of stability or impurities of the OA standards directly affect the quantification, either overestimating, in the case of degradation of the OA, or underestimating, when impurities that can inhibit the PP2A are present. Therefore, greater emphasis was put on these components and the ‘between batch homogeneity’ was evaluated besides the stability of the components. The between batch homogeneity was studied by taking 1 set of standards or 1 vial of phosphatase from 5 different batches. These batches were chosen along the estimated shelf life of the compounds and tested in one single assay together with internal control samples. All batches performed according to the assays’ specifications (*r*^2^ > 0.96) and the relative standard deviation was far below 15%, the expected value for samples assayed under repeatability conditions [19]. These results proved the stability of the enzyme for over 12 months at 4 °C and the homogeneity of between all batches tested (Table 2).

**Table 2:** Phosphatase stability and homogeneity. Five different phosphatase batches were tested at different stages of shelf life. Mean, standard deviation (SD) and relative standard deviation (RSDr) were calculated. Three internal control samples were used to verify correct quantification.

PP2A batch (shelf life)	Sample 1 (µg/kg)	Sample 2 (µg/kg)	Sample 3 (µg/kg)
1 (2 months)	95	160	310
2 (4 months)	100	169	304
3 (8 months)	88	162	323
4 (10 months)	94	156	300
5 (12 months)	90	144	341
mean	93	158	316
SD	5	9	17
RSDR	4.8%	6.0%	5.2%

For the OA standards, the same strategy was used. Five batches, covering 90% of the shelf life of the component (6 months), were tested in one assay to be able to single out the variation due to the standards’ stability and homogeneity (Table 3). A sample shown to be blank (0 nM) was included to be able to calculate the effect of variables other than OA. The RSDr calculated from the absorbance values were all <3%, proving the stability and homogeneity of the standards over 6 months.

**Table 3:** OA standards stability and homogeneity. Five different batches of OA standards were tested at different stages of shelf life. The absorbances (405 nm) obtained for each of the standards are shown. Mean, standard deviation (SD) and relative standard deviation (RSDr) of these absorbances were calculated.

Standards	Absorbance 405 nm							
	batch 1	batch 2	batch 3	batch 4	batch 5	mean	SD	RSDr
OA (nM)	5 months	4 months	3 months	2 months	1 week			
0.0	2.042	2.100	2.064	2.073	2.120	2.079	0.031	1.5%
0.5	1.622	1.614	1.649	1.625	1.678	1.637	0.026	1.6%
0.8	1.462	1.390	1.386	1.375	1.372	1.397	0.037	2.7%
1.2	1.124	1.116	1.101	1.092	1.134	1.113	0.017	1.5%
1.8	0.772	0.792	0.769	0.822	0.809	0.793	0.023	2.9%
2.8	0.619	0.646	0.606	0.637	0.613	0.624	0.017	2.7%

### 3.3. Ruggedness

Enzymatic assays, such as OkaTest, can be sensitive to environmental factors, such as temperature, incubation time or reagent volume. To determine the impact of these factors, samples with concentrations around the regulatory limit were quantified at normal and suboptimal conditions (Table 4). The effect of temperature was tested by performing the OkaTest assay at three different temperatures 28, 30 and 32 °C, obtaining a RSD of 1.0%. These results showed that temperature variations of 2 °C did not affect the performance as RSDr values were lower than 10% usually obtained in the assay (Table 5). 

Duration and pipetting volumes were evaluated alike and none of the variables affected the results of the test, with the exception of large pipetting errors. Pipetting errors of 5 µL in samples or phosphatase addition (errors of 10% and 7.1%, respectively) gave RSDr values of 14% and 17%, respectively. Precision in substrate addition was less critical. Pipetting samples and phosphatase are, however, the main sources of variability affecting PPIA and therefore care should be taken when adding these components.

**Table 4:** Ruggedness testing. The effects of variations of the normal assay conditions on sample quantification are shown.

Variable	Normal value	Variation	Mean value (µg/kg)	RSDr
Temperature	30 °C	±2 °C	175	1.0%
Pre-incubation	20 min	18, 20, 22, 24 min	158	3.6%
Incubation	30 min	27, 30, 33, 36 min	147	2.9%
Syst. pipetting error	50, 70, 90 µL	±2 µL	155	4.3%
Random pipetting error				
Sample	50 µL	±5 µL	151	14%
PP2A	70 µL	±5 µL	153	17%
Substrate	90 µL	±5 µL	158	6.1%
Phosphatase solubility time	60 ± 5 min	±30 min	158	5.0%

**Table 5:** Intermediate precision of ten different mussel and scallops samples. Mean, standard deviation (SD), relative standard deviation (RSDr) were calculated. < 63: below the working range of the assay (63–352 µg/kg).

Sample	Origin	Day 1 (µg/kg)	Day 2 (µg/kg)	Day 3 (µg/kg)	Mean	SD	RSDr
1	Mussel	211	227	187	208	20	9.5%
2	Mussel	122	132	113	122	10	7.8%
3	Scallop	<63	<63	<63	-	-	-
4	Mussel	82	94	90	88	6	7.0%
5	Mussel	196	196	215	202	11	5.2%
6	Scallop	<63	<63	<63	-	-	-
7	Mussel	<63	<63	<63	-	-	-
8	Scallop	125	108	117	117	8	7.0%
9	Mussel	250	253	281	261	17	6.5%
10	Mussel	277	279	289	282	7	2.4%

### 3.4. Applicability

**Figure 3:**
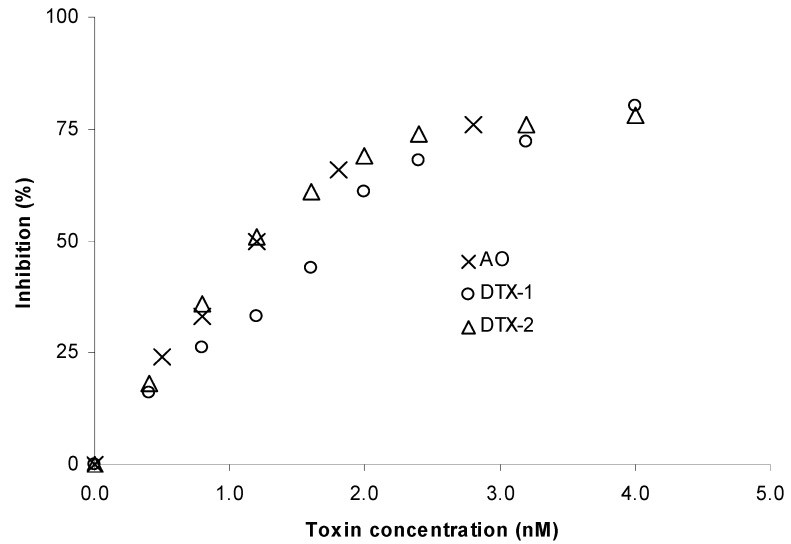
Phosphatase inhibition curve obtained with okadaic acid (OA), dinophysistoxin-1 (DTX-1) and dinophysistoxin-2 (DTX-2). Each point is the mean obtained from three different phosphatase batches. The standard deviation is not shown to maintain the figure legible. The IC_50_ values were 1.2 nM for both OA and DTX-2, and 1.6 nM for DTX-1.

There are numerous descriptions of the application of protein phosphatase inhibition assays for determination of OA and its derivatives [7,8,9,10,11,12,13]. However, the inhibition pattern of OA, DTX1 and DTX2 is different and is supposed to correspond to their toxicity. One way to evaluate the inhibition capacity of toxins on an enzyme is by determining the IC_50_, the concentration of toxin able to inhibit 50% of the maximum enzyme activity. This concentration depends, among others, on the amount of enzyme and the substrate concentration present in the assay [20] and therefore the IC_50_ values published for these toxins are difficult to compare [7,8,12,18,21,22]. The IC_50_ values found in our study were 1.2 nM for both OA and DTX-2, and 1.6 nM for DTX-1 (Figure 3) and are in accordance with the ones obtained recently by Huhn *et al*., 2009 [21]. However, these do not exactly correspond to the toxicity factors (TEF) that are used in analytical methods such as LC-MS/MS; as OA and DTX-1 have a TEF of 1, while DTX-2 has a TEF of 0.6, indicating equal toxicity for DTX-1 and OA and less toxicity for DTX-2 [2]. According to these values, our results would lead to an overestimation of the amount of DTX-2 and an underestimation of the amount of DTX-1 when compared with methods such as LC-MS/MS. However, the recovery data obtained for both DTX-1 and DTX-2 were similar to the ones obtained for OA (Table 6) suggesting that difference has a low impact in the determination of the level of toxins in shellfish samples.

**Table 6:** Recovery of the different toxins was calculated testing 5 samples at 0.5, 1 and 1.5 times the regulatory limit on 3 different days. OA Certified Reference Material (NRC CRM-OA-c) was spiked on mussel and king scallop. DTX-1 and DTX-2 were spiked on king scallop. ND: not determined.

Toxin	Matrix	Recovery (RSDr)		
		80 µg/Kg	160 µg/Kg	240 µg/Kg
OA	Mussel	101% (15%)	90% (8.9%)	78% (5.4%)
	King scallop	114% (9.9%)	98% (8.4%)	106% (8.7%)
DTX-1	King scallop	102% (15%)	79% (12%)	88% (17%)
DTX-2	King scallop	93% (2.3%)	ND	ND

### 3.5. Limit of Detection, Limit of Quantification, Repeatability and Reproducibility

The limit of detection (LOD) and limit of quantification (LOQ) were determined by using a blank +3 SD or blank +10 SD approach [14]. For blank mussel material, the LOD and LOQ were 44 and 56 µg/kg, respectively. These values are both below the working range of the test and sufficiently below the current European legal limit of 160 µg/kg. 

To estimate the precision, the assay was tested both under repeatability and intermediate precision conditions. The repeatability characteristics were estimated by analyzing 8 fractions of two naturally contaminated mussel samples and RSDr of 1.4% with a mean of 276 µg/kg, and 3.9% with a mean of 124 µg/kg were obtained (results not shown). The intermediate precision of the test was estimated by analyzing 7 samples with OA-toxin levels covering the working range of the assay on three different days by the same analyst. For all samples, the RSDr was well below the 15% RSDr limit as calculated by Horwitz [19]. Three samples tested as negative by LC-MS/MS were included to evaluate the consistency of the negative results (Table 5). 

### 3.6. Accuracy

The accuracy of the method was estimated by calculating recoveries for OA, DTX-1 and DTX-2 and by testing a Certified Reference Material (NRC-CNRC). Five portions containing 5 grams of mussel or king scallop were spiked with one of the three toxins at 0.5, 1 and 1.5 times the regulatory limit (80, 160 and 240 µg/kg), except for DTX-2 that was only added up to a concentration of 80 µg/kg. The five portions were analysed on three different days to determine the intermediate precision characteristics of the test. OA recoveries between 78 and 101% in mussel and 98 and 114% in king scallop were obtained. RSDr values for this toxin were below or equal to 15%. Similar recoveries were obtained for the other two toxins (Table 6). These recoveries are in agreement with the 75 to 120% range that is expected for this concentration range [19]. The RSDr results in this study were higher than the ones obtained in the precision experiments (Table 4), specially for DTX-1. This might be a consequence of the spiking. As mentioned before, the higher IC_50_ for DTX-1 compared to OA and DTX-2 had a low impact on the recovery.

Finally, four aliquotes of blank samples were spiked with the Certified Reference Material. The methanolic extract obtained was analysed with and without hydrolysis, and the recovery was estimated using the DTX-1 and OA content reported for the certified material. The recovery for the non-hydrolysed samples ranged from 71% to 98%, with a mean of 87% for mussle and 91% for king scallop (Table 7). These are acceptable recoveries and in accordance with the results showed in Table 6. However, the mean recovery of the hydrolysed samples was a 146% and 163% for mussle and king scallop, respectively. These percentages were far above the expected content of OA-toxins indicated in the reference material [23]. This could be due to the fact that the material is only certified for OA and DTX-1. Other esters of OA and DTX are reported in the certificate of anlaysis for this material.

**Table 7:** Recovery experiment with Certified Reference Material (NRC CRM-DSP-MUS-b). Samples were analysed with and without hydrolysis.

		Without hydrolysis		With hydrolysis	
Matrix	Spiked level (µg/kg) (*n*)	Recovery	RSDr	Recovery	RSDr
mussel	219 (4)	87%	14%	146%	12%
king scallop	180 (4)	91%	5.0%	163%	2.8%

### 3.7. Method Comparison

A method comparison among MBA, LC-MS/MS and OkaTest was performed with a total of 37 samples. Results were compared qualitatively for all three methods and quantitatively between OkaTest and LC-MS/MS. The 160 µg/kg regulatory limit was used to decide whether the samples were positive or negative (Table 8). 

**Table 8:** Methods comparison. Results from OkaTest, MBA and LC-MS/MS. 31 of the 37 samples were tested by MBA. Positive results (+): ≥160 µk/kg. Negative results (-): <160 µg/kg. LOQ. Limit of quantification. NA: not available.

ID	M	MBA	LC-MS/MS	OKATEST	LC-MS/MS	OKATEST
1	**Cockle**	-	-	-	<LOQ	<LOQ
2	**Cockle**	+	+	+	193	252
3	**Donax**	-	-	-	82	97
4	**Mussel**	+	+	+	502	232
5	**Mussel**	+	-	+	<LOQ	268
6	**Mussel**	+	+	+	604	>352
7	**Mussel**	+	+	+	894	>352
8	**Mussel**	+	+	+	414	306
9	**Mussel**	+	+	+	444	>352
10	**Mussel**	NA	-	-	<LOQ	<LOQ
11	**Mussel**	NA	+	+	357	>352
12	**Mussel**	NA	-	-	<LOQ	<LOQ
13	**Mussel**	NA	-	-	<LOQ	<LOQ
14	**Mussel**	-	-	-	<LOQ	122
15	**Mussel**	+	-	+	158	196
16	**Mussel**	+	+	+	177	250
17	**Mussel**	+	+	+	288	265
18	**Mussel**	+	+	+	202	196
19	**Mussel**	+	+	+	390	277
20	**Mussel**	+	+	+	658	305
21	**Mussel**	+	+	+	392	310
22	**Mussel**	+	+	+	329	315
23	**Mussel**	+	+	+	232	270
24	**Mussel**	+	+	+	235	277
25	**Mussel**	+	-	-	152	135
26	**Mussel**	+	-	+	98	164
27	**Mussel**	+	+	+	168	211
28	**Mussel**	+	+	+	209	251
29	**Mussel**	+	-	+	113	191
30	**Mussel**	NA	+	-	292	<LOQ
31	**Mussel**	NA	+	+	316	304
32	**Mussel**	-	-	-	<LOQ	<LOQ
33	**Mussel**	+	+	-	177	124
34	**Mussel**	+	+	+	247	216
35	**Mussel**	+	+	-	185	144
36	**Scallop**	+	+	+	184	264
37	**Scallop**	-	-	-	<LOQ	<LOQ

In general, the qualitative interpretation of the results indicates that the three methods obtained equivalent results, especially taking into account that these are conceptually different methods. The OkaTest disagreed with both MBA and LC-MS/MS on two occasions (samples 33 and 35). OkaTest detected levels of OA-toxins in those two samples, but below the EU regulatory limit (124 and 144 µg/kg), while the samples were positive according to the other two methods). A third sample (25) was also identified as negative by OkaTest and positive by MBA. LC-MS/MS also gave a negative result for sample 25. The concentration of this sample determined by both methods was just below the EU regulatory limit. 

The LC-MS/MS differed on four occasions: all four negative according to LC-MS/MS, but positive by the other two methods. Three of the samples (15, 26 and 29) contained OA-toxins below the EU refulatory limit, but sample 5 was quantified under the method’s LOQ. Finally, one sample (30) was positive by LC-MS/MS, but under the LOQ by OkaTest. Sample 30 was not tested by MBA due to lack of material. 

Quantitative results obtained by LC-MS/MS and Okatest showed some differencies. About two thirds of the samples gave similar results (±25%) with both methods, but the rest of the samples did not show a clear tendency. There is no evident explanation for this and further investigation would be required.

## 4. Conclusions

A colorimetric phosphatase inhibition assay for determination of OA-toxins, OkaTest, was single laboratory validated according to international methods validation guidelines. The limit of quantification of the method is well below the EU regulatory limit and the method permitted the easy quantification of up to 43 samples within one hour, excluding sample preparation. The method is robust, with very good precision characteristics, adequate specificity and accuracy. 

This colorimetric phosphatase inhibition assay could be used as a complementary assay to the reference method for determination of lipophilic toxins, once a collaborative study has been completed and it has been successfully tested under recognized proficiency tests. This assay could be applied for monitoring purposes when OA-toxins are identified to be responsible for a bloom. 
